# Multiple plant growth-promoting bacteria enhance rice growth in non-grain-converted lands

**DOI:** 10.3389/fbioe.2026.1792606

**Published:** 2026-03-25

**Authors:** Xuqing Li, Wu Ying, Munazza Ijaz, Temoor Ahmed, Muhammad Shafiq Shahid, Gabrijel Ondrasek, Branko Petrinec, Jianli Yan, Zhongling Tian, Bin Li

**Affiliations:** 1 Institute of Vegetable, Hangzhou Academy of Agricultural Sciences, Hangzhou, China; 2 State Key Laboratory of Rice Biology and Breeding, Ministry of Agriculture and Rural Affairs Key Laboratory of Molecular Biology of Crop Pathogens and Insect Pests, Zhejiang Provincial Key Laboratory of Agricultural Microbiomics, Institute of Biotechnology, Zhejiang University, Hangzhou, China; 3 Department of Plant Biotechnology, Korea Universtiy, Seoul, Republic of Korea; 4 Department of Plant Sciences, College of Agricultural and Marine Sciences, Sultan Qaboos University, Muscat, Oman; 5 Faculty of Agriculture, University of Zagreb, Zagreb, Croatia; 6 Institute for Medical Research and Occupational Health, Zagreb, Croatia; 7 Faculty of Dental Medicine and Health, Josip Juraj Strossmayer University of Osijek, Osijek, Croatia; 8 Key Laboratory of Pollution Exposure and Health Intervention of Zhejiang Province, Interdisciplinary Research Academy, Zhejiang Shuren University, Hangzhou, China

**Keywords:** identification, microbial community, non-grain-converted land, plant growth-promoting bacteria, plant growth-promoting traits, soil properties

## Abstract

China faces a continuously growing food demand, while a large proportion of its cultivated land is increasingly being shifted to non-grain plantation, leading to widespread soil fertility degradation and microbial community imbalance in lands converted back to grain production. To address the critical research gap of targeted soil fertility restoration and yield enhancement in these marginal non-grain-converted cultivated lands using microbial-based strategies, this study aimed to screen and identify plant growth-promoting bacteria (PGPB) and systematically assess their effects on soil health and rice growth in non-grain-converted fields. Bacteria were isolated from the rhizosphere soils of non-grain-converted fields and identified through morphology and multilocus gene sequencing. Key plant growth-promoting (PGP) traits, including phosphate solubilization, nitrogen fixation, siderophore formation, and indole-3-acetic acid (IAA) production, were assessed. The effects of these strains on soil microbial communities and soil properties in converted rice fields were further evaluated through pot experiments and high-throughput sequencing. Among 589 isolated bacterial strains, eight were screened out with robust PGP traits, including phosphate solubilization capacity (solubilization zone diameter: 11.74–24.82 mm), siderophore production (orange zone diameter: 8.28–10.57 mm), IAA synthesis (25.61–96.22 μg/mL) and nitrogen fixation capacity. *In vivo* pot assays showed that three elite strains (LA-B511, YH-S3, and LA-B111) significantly promoted rice seedling growth, leading to increases in seedling height by 25.28%, 24.90%, and 18.86%; root length by 16.81%, 13.82%, and 21.95%; seedling dry weight by 20.81%, 38.55%, and 33.78%; and root dry weight by 27.17%, 25.74%, and 50.84%, respectively. Morphological and molecular analyses identified these three strains as *Enterobacter hormaechei* and *Yokenella regensburgei*. After 35 days of inoculation, soil available phosphorus (AP) content increased by 27.00%, 25.99%, and 16.65% compared to the non-inoculated control. Additionally, soil microbial communities were significantly reshaped, driven by changes in soil organic matter (SOM), soil pH, iron (Fe) content, total phosphorus (TP) and available phosphorus (AP). Overall, our results demonstrated that the screened PGPB strains can effectively improve rice seedling growth and soil health in non-grain-converted cultivated lands, providing a promising microbial-based amendment for early-stage rice establishment and sustainable grain production potential in these specific marginal non-grain-converted cultivated lands.

## Introduction

Food security is the foundation of any country, and cultivated land is crucial for both food production and ecological stability (Jiang et al., 2024). However, because humans rely heavily on cultivated land for survival, there has been a significant increase in the conversion of farmland to non-grain uses in past years (Chen et al., 2023). Therefore, safeguarding and effectively using cultivated land to secure food supply and achieve sustainable agricultural growth has become a critical strategic priority for China’s economic progress, national security and social stability. China attributes great importance to the issues of “non-grain cultivation” and food security. However, due to various factors such as increased urbanization, economic development, and dietary structure changes, many agricultural entities prioritize profit maximization, leading to a significant shift of grain-cultivated land to economically valuable non-grain plants (including tea, vegetables, fruits, flowers and bamboo shoots) in recent years (Chen et al., 2023; Li and Hao, 2023). These non-grain crops often rely on excessive chemical inputs (particularly chemical nitrogen fertilizers), which has resulted in severe soil degradation, including fertility decline, acidification, topsoil structure damage, and disrupted native soil microbial balance (Boregowda et al., 2022; He et al., 2024; Shrivas et al., 2024). To address this situation and safeguard food security, Zhejiang Provincial Government has implemented targeted measures to revert non-grain-cultivated lands back to grain production, with a focus on rice (the dominant grain crop in Zhejiang, accounting for over 70% of the province’s total grain output) (Zhejiang Provincial Bureau of Statistics, 2023). Consequently, restoring degraded soil and improving grain productivity in these converted lands has become an urgent practical challenge.

Soil microorganisms are core drivers of healthy soil ecosystems, playing a decisive role in regulating soil fertility, crop yield, and stress resilience in agroecosystems (Hartmann and Six, 2023; Romão et al., 2025). Among diverse functional soil microbes, beneficial bacteria known as PGPB have emerged as a promising green alternative to chemical fertilizers in sustainable agriculture (Wang et al., 2023). A wide range of PGPB genera, including *Agromyces*, *Arthrobacter*, *Azotobacter*, *Bacillus*, *Burkholderia*, *Enterobacter*, *Pantoea*, *Paenibacillus*, *Pseudomonas*, *Streptomyces* etc., have been extensively studied (Bukhat et al., 2020; Anzalone et al., 2021; Christakis et al., 2021; Li et al., 2021; Pérez et al., 2023; Flores et al., 2025). These beneficial bacteria can establish their colonies in plant roots and boost crop productivity through multiple mechanisms, including enhancing soil nutrient accessibility, bio-fixing nitrogen, solubilizing soil minerals, enhancing iron uptake, minimizing chemical fertilizer use, producing phytohormones, and improving resistance against both biotic and abiotic stress (Armada et al., 2014; Kumar and Verma, 2018; Lopes et al., 2021; Cheng et al., 2025; Kiprotich et al., 2025; Zhao et al., 2025). To date, some elite PGPB strains have been effectively adopted as biofertilizers and biostimulants in agricultural systems (Ruiu, 2020; Attia et al., 2022). However, although non-grain-to-grain conversion drastically alters soil physicochemical properties and microbial communities (Li et al., 2025), few studies have focused on microbial diversity dynamics in these converted lands (particularly the community composition and functional potential of native PGPB), or revealed how native PGPB adapt to degraded soil environments and regulate microbial communities to facilitate soil restoration (Chen et al., 2024). Given the complexity of PGPB-mediated plant growth promotion and soil quality enhancement (Poria et al., 2022), screening novel native PGPB strains with numerous strong PGP traits is very important for the remediation of non-grain-converted soils and the enhancement of crop productivity.

In addition to this, PGPB can also indirectly benefit plants by influencing soil properties and modulating native microbiota (Yan et al., 2024; Lahijanian et al., 2025). For instance, Khoso et al. (2024) demonstrated that PGPB could improve soil texture by regulating extracellular molecule secretion and signal transduction, while Xie et al. (2024) found that salt-tolerant PGPB could enhance soil structure and porosity by producing exopolysaccharides during metabolism. Moreover, PGPB inoculation can also reshape the rhizosphere microbiome, enhance microbial diversity, enrich beneficial functional microbes, and strengthen antagonism against soil-borne pathogens (Wang et al., 2022; Yan et al., 2024), thereby underpinning soil structure, fertility, and ecosystem resilience (Ren et al., 2021; Kiprotich et al., 2025). Soil microbial communities are widely recognized as fundamental to maintaining soil health, ecosystem functions, and long-term sustainable productivity of cultivated land (Naylor et al., 2022; Banerjee and van der Heijden, 2023), but they are highly sensitive to changes in land use patterns, agri-management practices, and plant varieties (Schloter et al., 2018; Singh et al., 2021). Long-term non-grain cash crop planting severely disrupts soil microbial community structure, reduces microbial diversity, and impairs the ecological functions of the soil microbiome (Li B. et al., 2024; Dunn et al., 2025). Furthermore, the conversion from upland non-grain fields to paddy fields further alters soil physicochemical properties and nitrogen-related microbial functions across various soil depths (Li B. et al., 2024). Despite these findings, critical knowledge gaps still exist regarding the application of PGPB in non-grain-converted lands. Most current PGPB strains are isolated from healthy conventional farmland, whereas native PGPB adapted to the degraded soil environment of non-grain-converted lands remain largely unexplored (Chen et al., 2024). Accordingly, the screening of functional native PGPB and the underlying mechanisms by which PGPB inoculation reshapes rhizosphere microbial communities and maintains soil health in converted fields have not been systematically elucidated, severely restricting the development and application of targeted microbial amendments for non-grain-converted cultivated lands.

In the present study, we proposed two scientific hypotheses: (1) native PGPB strains isolated from the rhizosphere of non-grain cultivated lands can adapt well to the degraded soil environment of non-grain-converted rice fields and exert excellent PGP effects on rice; (2) inoculation with these native PGPB can improve soil health in non-grain-converted rice fields by improving soil physicochemical properties and reshaping rhizosphere microbial community structure. Thus, the aims of this study were to isolate and screen native bacterial strains with multiple PGP traits (including phosphate solubilization, nitrogen fixation, siderophore production, and IAA synthesis) from rhizosphere soils of non-grain cultivated lands; to identify elite strains through morphological characterization and molecular sequence analysis (16S *rRNA*, *gyr*B, *inf*B, *rpo*B and *atp*D); to evaluate the bioremediation potential of these elite PGPB by verifying their effects on rice growth, soil physicochemical properties, and rhizosphere microbial communities in non-grain-converted rice fields through pot experiments.

## Materials and methods

### Soil sample collection

A total of 108 soil samples were obtained from six independent sampling sites with diverse land-use conversion modes in Zhejiang Province, China: (1) conversion of loquat orchard to rice field in Jiande (29°20′6″N; 119°29′38″E); (2) conversion of mulberry field to rice field in Chun’an (29°29′42″N; 118°36′12″E); (3) conversion of blueberry orchard to rice field in Tonglu (29°46′42″N; 119°20′8″E); (4) conversion of vineyard to rice field in Fuyang (30°3′13″N; 120°1′9″E); (5) conversion of bamboo garden to rice field in Lin’an (30°6′25″N; 119°44′4″E); and (6) conversion of nursery to rice field in Yuhang (30°28′5″N; 120°8′25″E). Basic soil physicochemical properties of the six sampling sites were as follows: pH 4.76–7.17, SOM 11.27–50.98 g/kg, and AP 5.90–165.10 mg/kg. At each sampling site, eighteen soil samples were obtained, comprising six biological replicates from non-grain-converted land, six biological replicates from adjacent rice fields transformed from the corresponding non-grain-converted land, and six biological replicates from local perennial paddy fields. All samples were taken at 5–20 cm depth around the rhizosphere by using five-point sampling technique (Panagos et al., 2014). Samples were then transported to the laboratory with caution for subsequent analysis.

### Bacterial isolation and identification

20 g of fresh soil were suspended in 180 mL of sterile distilled water and thoroughly mixed by vortexing for 10 min. The soil suspension was serially diluted in sterile distilled water using 9 mL dilution blanks. From the 10^–4^ dilution, 100 μL was aseptically transferred and evenly distributed on Luria-Bertani agar medium (LA; peptone 10 g, NaCl 10 g, yeast extract 5 g, agar 20 g, distilled water to 1000 mL; pH 7.2–7.5). The plates were placed at 28 °C for 48 h in an incubator. After purification by repeated streaking on LA medium (three consecutive streaks to ensure purity), bacterial colonies with distinct morphologies (diverse sizes, shapes, colors, and textures) were chosen and preserved in 30% (v/v) glycerol at −70 °C for long-term storage.

Identification of the bacterial strains was carried out by amplifying the 16S *rRNA* gene using polymerase chain reaction (PCR). For this, PCR reaction mixture (50 μL total volume) was prepared with the following components: 2×Hieff® PCR Master Mix (25 μL), 10 μM “27F (forward primer, 5′-AGA GTT TGA TCC TGG CTC AG-3′, 2 μL)”, 10 μM “1492R (reverse primer, 5′-GGT TAC CTT GTT ACG ACT T-3′, 2 μL)”, ddH_2_O (18 μL), and DNA (3 μL) (Eswaran and Khandeparker, 2017). PCR was performed under the following cycling conditions: an initial denaturation at 94 °C for 5 min; 35 cycles of denaturation at 94 °C for 30 s, annealing at 53 °C for 30 s, and extension at 72 °C for 60 s; followed by a final extension at 72 °C for 10 min. PCR amplified products were visualized on 1.0% agarose gels with ethidium bromide, then sent to Tsingke Biotechnology Co., Ltd. (Hangzhou, China) for Sanger sequencing. Using BLASTn, the resulting sequences were preliminarily assigned to different genera through sequence similarity searching in GenBank database. Furthermore, the identity of *Enterobacter* sp. and *Pantoea* sp. was assayed by amplifying housekeeping genes (including *gyr*B, *inf*B, *rpo*B, and *atp*D) using primer sets “*gyr*B-F/*gyr*B-R (5′-CGA CAA CTC GAT CGA CGA-3′ and 5′-GAC AGC AGC TTG TCG TAG-3′)”, “*inf*B01-F/*inf*B02-R (5′-ATY ATG GGH CAY GTH GAY CA-3′ and 5′-ACK GAG TAR TAA CGC AGA TCC A-3′)”, “*rpo*BCM-F/*rpo*BCM31b-R (5′-AAC CAG TTC CGC GTT GGC CTG-3′ and 5′-CCT GAA CAA CAC GCT CGG A-3′)”, “*atp*D01-F/*atp*D02-R (5′-RTA ATY GGM GCS GTR GTN GAY GT-3′ and 5′-TCA TCC GCM GGW ACR TAW AYN GCC TG-3′)”, respectively, whereas *Pseudomonas* sp. and *Yokenella* sp. were assessed by amplifying *gyr*B and *rpo*B. The PCR reaction volume for housekeeping genes was 50 μL, consistent with the protocol described for the 16S *rRNA* gene. The amplification conditions for *infB*, *rpoB*, and *atpD* genes were: an initial denaturation at 94 °C for 5 min; 35 cycles of denaturation at 94 °C for 30 s, annealing at 55 °C for 30 s, and extension at 72 °C for 60 s; followed by a final extension at 72 °C for 10 min. In contrast, amplification of the *gyrB* gene was performed with an initial denaturation at 95 °C for 5 min; 30 cycles of denaturation at 95 °C for 60 s, annealing at 54 °C for 60 s, and extension at 72 °C for 120 s; followed by a final extension at 72 °C for 5 min. After sequencing, PCR products from each region were assembled and edited using DNASTAR Lasergene (v7.1.0) to remove low-quality sequences and adaptors. The resulting high-quality sequences were compared with sequences present in GenBank by using BLASTn. Isolates were identified at the genus level with ≥95% sequence similarity and at the species level with ≥99% similarity. All gene sequences (16S rRNA, *gyrB*, *infB*, *rpoB*, *atpD*) were deposited in the GenBank, with accession numbers provided in Table 1. Closely related reference sequences were retrieved from GenBank based on BLASTn results. Maximum-likelihood (ML) phylogenetic trees were constructed using MEGA 7.0 software (Kumar et al., 2016), based on the Kimura 2-parameter (K2P) model, with 1000 bootstrap replicates to assess branch support.

**TABLE 1:** Bacterial sequences used in the current study.

Species	Strains	GenBank accession number				
		16S *rRNA*	*gyr*B	*rpo*B	*inf*B	*atp*D
*Enterobacter asburiae*	CCGL988	ON999069	OP006448	OP006450	OP542231	OP006449
	**CA-M1130**	**PQ269751**	**PQ516239**	**PQ306058**	**PQ282442**	**PQ282431**
*E. cancerogenus*	LMG2693	NR_044977	FJ617354	JX425239	JX425109	JX424850
*E. chuandaensis*	090028^T^	NR_180237	MK056241	MK056243	MK056244	MK056246
*E. cloacae*	ATCC13047^T^	NR_102794	EU643470	EU643264	JX425106	JX424847
*E. hormaechei*	CCUG26643	OR825716	JX424984	JX425243	JX425113	JX424854
	**LA-B111**	**PQ269753**	**PQ516241**	**PQ306060**	**PQ282444**	**PQ282433**
	**LA-B511**	**PQ269754**	**PQ516242**	**PQ306061**	**PQ282445**	**PQ282434**
*E. huaxiensis*	090008^T^	NR_180236	MK056248	MK056242	MK056245	MK056247
*E. kobei*	CCUG49023 = EK9	MZ317532	JX494750	JX494753	JX494751	JX494748
*E. ludwigii*	LMG23768	NR_042349	JX424985	JX425244	JX425114	JX424855
*E. mori*	LMG25706^T^	MG846019	JX424992	JX425251	JX425121	JX424862
*E. pseudoroggenkampii*	FY158	CP129026	CP129026	CP129026	CP129026	CP129026
	**YH-S-R21**	**PQ269759**	**PQ516247**	**PQ306066**	**PQ282450**	**PQ282439**
*E. sichuanensis*	WCHECL1597^T^	MG832788	MG832789	MG832790	MH513960	MH513959
*E. soli*	LMG26282	MN075257	JX424995	JX425254	JX425124	JX424865
	**FY-R811**	**PQ269760**	**PQ516248**	**PQ306067**	**PQ282451**	**PQ282440**
*E. wuhouensis*	WCHEs120002	MK567957	MK613100	MK613102	MK613101	MK613099
*Pantoea agglomerans*	LMG1286	FJ611839	EF988798	EF988970	EF988884	EF988711
*P. ananatis*	LMG2665	Z96081	FJ617371	KF482748	EF988910	EF988737
	**CA-R211**	**PQ269761**	**PQ516249**	**PQ306068**	**PQ282452**	**PQ282441**
*P. anthophila*	LMG2558	NR_116113	EF988812	EF988984	EF988898	EF988725
*P. brenneri*	LMG5343	EU216735	EU145270	EU145302	EU145286	EU145254
*P. calida*	140007	GQ367478	GQ367480	GQ367479	GQ367476	GQ367477
*P. cdeleyi*	LMG24200	EF688011	EF988770	EF988942	EF988856	EF988683
*P. citrea*	LMG22049	EF688008	EF988802	EF988974	EF988888	EF988715
*P. conspicua*	LMG24534	EU216737	EU145269	EU145301	EU145285	EU145253
*P. cypripedii*	LMG2657	NR_119369	FJ187830	FJ187840	FJ187835	FJ187825
*P. dispersa*	LMG2603	DQ504305	EF988818	EF988990	EF988904	EF988731
*P. eucalypti*	LMG24197	NR_116112	EF988762	EF988934	EF988848	EF988675
*P. eucrina*	LMG2781	EU216736	EU145271	EU145303	EU145287	EU145255
*P. gaviniae*	A1807	GQ367483	GQ367485	GQ367484	GQ367481	GQ367482
*P. punctata*	LMG22050	EF688006	EF988803	EF988975	EF988889	EF988716
*P. rodasii*	LMG26273	JF295053	JF295023	JF295043	JF295033	JF295013
*P. rwandensis*	LMG26275	JF295055	JF295030	JF295050	JF295040	JF295020
*P. septica*	LMG5345	EU216734	EU145272	EU145304	EU145288	EU145256
*P. stewartii*	LMG2715	Z96080	EF988831	EF989003	EF988917	EF988744
*P. terrea*	LMG22051	EF688007	EF988804	EF988976	EF988890	EF988717
*P. vagans*	LMG24199	EF688012	EF988768	EF988940	EF988854	EF988681
*P. wallisii*	LMG26277	JF295057	JF295031	JF295051	JF295041	JF295021
*Pseudomonas aeruginosa*	SC-1	FJ652616	FJ652724	FJ652697	**–**	**–**
*P. brassicacearum*	Wood1	KT695843	KX696680	KX696905	**–**	**–**
*P. chlororaphis*	L5737	NR_116763	CP083442	FJ652691	**–**	**–**
*P. fluorescens*	2P24	AY447045	KT020758	KU963679	**–**	**–**
*P. koreensis*	PF35	MF838692	MG021897	MG021969	**–**	**–**
*P. mediterranea*	UYT92018	PP066855	OL862564	OL862530	**–**	**–**
*P. mosselii*	NN10	OM022022	CP133092	OM202622	**–**	**–**
	**JD-L31**	**PQ269765**	**PQ516253**	**PQ306072**	**–**	**–**
*P. protegens*	EMM-1	MN959751	MT798861	MT799749	**–**	**–**
*P. putida*	LMG1246	HE586397	HF545879	AJ717485	**–**	**–**
*P. simiae*	25L1A	MT878378	MT941337	MT941383	**–**	**–**
*P. stutzeri*	A563/77	HF571102	HF571085	AJ864839	**–**	**–**
*P. synxantha*	S64109	KC834375	KC834147	KC834223	**–**	**–**
*P. syringae*	UMAF2811	NR_043716	JX867866	MH479224	**–**	**–**
*Yokenella regensburgei*	JCM3961	LC420110	CP050811	JX425344	**–**	**–**
	**YH-S3**	**PQ269768**	**PQ516256**	**PQ306075**	**–**	**–**
*Pectobacterium carotovorum*	LMG2404	NR_119367	KJ818410	HM359006	–	–

Sequences of bacterial strains in bold were obtained in this study, while the other bacterial sequences were obtained from GenBank database. ATCC13047^T^ = LMG2783; CCUG26643 = ZHB-3; LMG23768 = EN-119^T^; LMG26282 = JL8; L5737 = ATCC9446^T^; 2P24 = A11; UYT92018 = PVCT3C; NN10 = JP2-207; A563/77 = LMG6397; UMAF2811 = NCPPB281; JCM3961 = W13.

### Bioassays for PGP traits

All PGP trait assays were performed in three independent biological replicates, with three technical replicates per biological replicate, to ensure the reliability and reproducibility of results. Elite strains were screened via a stepwise strategy with the following quantitative criteria: phosphate solubilization zone diameter ≥11 mm; siderophore production orange halo diameter ≥8 mm; positive nitrogen-fixing activity (clear color change from green to blue); IAA synthesis yield ≥65 μg/mL in LB broth supplemented with 1.0% tryptophan. Only strains that satisfied most of the above criteria were selected for subsequent pot experiments, which are useful for controlled evaluations to minimize environmental heterogeneity (e.g., uneven soil fertility, variable climate, and complex agronomic management).

Phosphorus solubilization: the phosphorus-solubilizing capacity of the isolated strains was assessed by inoculating bacterial cultures on National Botanical Research Institute’s phosphate (NBRIP) media (Abdallah et al., 2019). Briefly, 10 μL of the bacterial suspension (approximately 10^7^ CFU/mL) was applied to a sterile 6-mm filter paper disc, which was then positioned at the center of NBRIP agar plates and incubated at 28 °C for 3 days. The ability of the isolates to solubilize phosphorus was determined by the presence of a transparent halo zone around the growth. Following the measurement of phosphorus-solubilizing zone size, the phosphorus solubilization index (PSI) was calculated using the formula: “PSI (%) = (halo zone diameter - colony diameter)/colony diameter × 100” to further assess the phosphorus-solubilizing ability.

Siderophore production: siderophore-producing capacity was evaluated by assay of chrome azurol S (CAS) (Murugappan et al., 2011). In this assay, solution A (CAS 0.0605 g, ddH_2_O 50 mL, 1 mM FeCl_3_·6H_2_O with 10 mM HCl 10 mL), solution B (Hexadecyltrimethylammonium bromide 0.0729 g, ddH_2_O 40 mL) and solution C (10x MM9 salts (KH_2_PO_4_ 0.3 g, NaCl 0.5 g, NH_4_Cl 1.0 g, NaOH 6.0 g, PIPEs 30.24 g, ddH_2_O 1000 mL) 100 mL, PIPEs 30.24 g, agar 15 g, ddH_2_O 750 mL, pH 6.8) were mixed with 10% casamino acids (30 mL), 20% glucose (10 mL), 1 M MgCl_2_ (1 mL), 100 mM CaCl_2_ (1 mL) by slowly adding into the mixture. For the CAS assay, 10 μL of the bacterial suspension (approximately 10^7^ CFU/mL) was applied to a sterile 6-mm filter paper disc, which was placed at the center of CAS agar plates and incubated at 28 °C for 3 days. Siderophore production was indicated by the development of an orange halo surrounding the colonies of siderophore producing bacteria.

Nitrogen fixation: bacterial strains with high phosphorus-solubilizing and siderophore-producing capacity were further analyzed for nitrogen-fixing ability. In brief, nitrogen-fixing ability was evaluated by inoculating 10 μL of bacterial suspension (approximately 10^7^ CFU/mL) into 5 mL of N-free malate broth (Nfb) containing KH_2_PO_4_ 0.4 g, K_2_HPO_4_ 0.1 g, MgSO_4_·7H_2_O 0.2 g, NaCl 0.1 g, CaCl_2_ 0.02 g, FeCl_3_ 0.01 g, Na_2_MoO_4_·2H_2_O 0.002 g, sodium malate 5.0 g, bromothymol blue in 0.5% alcohol 5 mL and distilled water 1000 mL, pH 7.0 (Tang et al., 2020). Cultures were incubated at 28 °C with shaking for 2 days at 180 rpm. Presence of nitrogen-fixing activity was indicated by change in color of medium from green to blue.

IAA production: to assess IAA production, 10 μL of bacterial suspension (approximately 10^7^ CFU/mL) was inoculated into 10 mL of LB broth (without agar) supplemented with either 1.0% or 0.1% (w/v) tryptophan, while LB media without adding tryptophan was used for control. Cultures were incubated at 28 °C with shaking at 180 rpm for 2 days. Then, 1.5 mL of each culture was collected and centrifuged at 8,000 rpm and 4 °C for 5 min. Subsequently, 1 mL of the supernatant was mixed with 4 mL of Salkowski’s reagent (H_2_SO_4_ 300 mL, 0.5 M FeCl_3_ 15 mL and distilled water 500 mL) (Li et al., 2021) and kept in dark for 20 min at 22 °C. IAA content was determined by change in color from pinkish to reddish and absorbance was determined at 530 nm via spectrophotometer (Perkin Elmer Lambda35, Waltham, MA, United States). The IAA concentration was then quantified based on the standard curve made from known IAA standards.

Plant growth promotion assays: to estimate the impact of the tested bacteria on rice, a pot-based experiment was conducted in a phytotron from April 29 to June 4, 2024, with a diurnal temperature of 25 °C day/18 °C night, a 16 h light/8 h dark photoperiod, and 70% relative humidity. Briefly, rice seeds (cultivar Xiushui 131) were obtained from the Jiaxing Academy of Agricultural Sciences. Surface sterilization was performed by immersing them in 75% (v/v) ethanol for 1 min, after which they were rinsed three times with sterile distilled water. These seeds were then placed on moist sterile filter paper and incubated at 25 °C for 2 days to allow germination. Subsequently, the sprouted seedlings were transplanted into plastic pots filled with approximately 1.0 kg of air-dried soil that had previously been used for vegetable cultivation. The soil had a pH of 7.83 and contained 7.78 g/kg SOM, 0.69 g/kg TP, 34.41 mg/kg AP, and 25.10 g/kg Fe. Each treatment consisted of nine pots with six uniform seedlings per pot, and three independent biological replicates were set for each treatment. All pots were arranged in a completely randomized design in the phytotron to eliminate positional effects. Two days after transplantation, 15 mL of each bacterial suspension (10^8^ CFU mL^-1^) were applied to the rhizospheric soil. While, control was irrigated with sterile double-distilled water. After 35 days of cultivation, the plants were carefully removed from the pots, washed with tap water and then their height, root length and the dry weights of both the seedlings and roots were recorded. In particular, dry weight was weighed after being dried in an oven for 24 h at 65 °C. To quantify the growth-promoting effect of the tested strains on rice, relative performance was expressed as growth promotion efficacy (GPE), calculated as a percentage according to the equation: “GPE (%) = (treatment - control)/control × 100”.

### Effects of PGPB on soil characteristics and microbes

After harvesting rice, approximately 0.5 kg soil was collected from all treatments to determine soil physicochemical properties. The samples were air-dried at room temperature, ground and passed through a 0.45-mm sieve. Subsequently, five soil properties (pH, SOM, TP, AP, and Fe) were analyzed following previously described methods. Briefly, pH of soil samples was determined in soil-water suspension (1:2.5, w/v) with pH meter (FE28, Mettler-Toledo, Zurich, Switzerland) (Qi et al., 2018). SOM was estimated *via* applying potassium dichromate volumetric-dilution heat process (Peng et al., 2024). TP content was analyzed by first subjecting samples to persulfate digestion and subsequently determining concentrations via automated colorimetric analysis (Stoddard et al., 2016), while AP was calculated by phosphomolybdate blue colorimetry (Zhan and Sun, 2011). Fe content was measured through the reduction of Fe^3+^ to Fe^2+^ by hydroxylamine HCl (Zhu et al., 2018). All assays were performed in triplicate.

At the same time, approximately 10 g of rhizosphere soil was collected from each treatment for microbial community analysis. The DNA was isolated from soil (1 g) using the E.Z.N.A.™ Mag-Bind Soil DNA Kit (Omega, United States), and DNA quality and concentration were assessed spectrophotometrically using a NanoDrop 1000 (Thermo Scientific, United States). High-throughput sequencing was performed by amplifying the ITS1 region of fungal ITS genes and the V3-V4 region of bacterial 16S *rRNA* genes through PCR using specific primers: “ITS1F (5′−CTT GGT CAT TTA GGA AGT AA−3′)” “ITS2R (5′−GCT GCG TTC TTC ATC GAT GC−3′)” (Adams et al., 2013) “341F (5′−CCT ACG GGN GGC WGC AG−3′)” and “806R (5′−GGA CTA CHV GGG TWT CTA AT−3′)” (Wu et al., 2015). The PCR reaction was performed following a standard protocol, and amplified PCR products were purified using Hieff NGS™ DNA Selection Beads (Yeasen, China) and subsequently combined in equimolar amounts. The pooled libraries were then subjected to 2 × 250 bp paired-end sequencing on an Illumina MiSeq platform (Ling’en Biotechnology Co., Ltd., Shanghai, China).

Bioinformatic analysis of sequencing data was performed according to the previously described methods (Li et al., 2023; Li B. et al., 2024; Tu et al., 2024). Briefly, paired-end reads were merged, quality-filtered and trimmed according to established protocols (Schmieder and Edwards, 2011; Zhang et al., 2014). High-quality clean reads sequences were then clustered into operational taxonomic units (OTUs) at 97% sequence similarity using USEARCH (v11.0.667) (Edgar, 2013; Edgar, 2016). Representative sequences for each OTU were selected using QIIME (v2020.06). Fungal and bacterial OTU representatives were then taxonomically assigned by BLAST searches against the UNITE and RDP databases, respectively, using a confidence threshold of 90% (Altschul et al., 1997; Wang et al., 2007; Caporaso et al., 2010; Quast et al., 2013; Urmas et al., 2013). Among these, the relative abundances (RAs) of *Yokenella* and *Enterobacter* were specifically analyzed and visualized using Origin software (v2023).

### Statistical analysis

The significance threshold for all statistical tests was set at *p* < 0.05. Following verification of normality and homogeneity of variance by the Shapiro–Wilk test and Levene’s test, respectively, one-way variance analysis (ANOVA) was conducted in SPSS (v16, SPSS, USA), and Duncan’s multiple range test was used for *post hoc* comparisons. Community richness and diversity were assessed using two alpha diversity indices, Chao1 and Shannon, which were analyzed and visualized with Origin software (v2023). Principal component analysis (PCA) based on Bray-Curtis beta diversity metrics was performed to compare community differences among different samples (Ramette, 2007). Statistical significance of community differences was evaluated using permutational multivariate ANOVA (PERMANOVA) with 999 permutations to compute *p*-values (Dixon, 2003). The community composition of dominant microbes at the phylum and genus levels was further analyzed and visualized by Origin software (v2023). In addition to this, linear discriminant analysis effect size (LEfSe) was employed to discover differential biomarkers between groups to reveal the intergroup differences among different samples (Segata et al., 2011). The influence of environmental factors on microbial community structure was assessed using redundancy analysis (RDA). Additionally, a correlation heatmap illustrating the relationships between soil physicochemical properties and microbial taxa was generated by R software (v4.1.3).

## Results

### Multifarious plant growth-promoting traits

**FIGURE 1:**
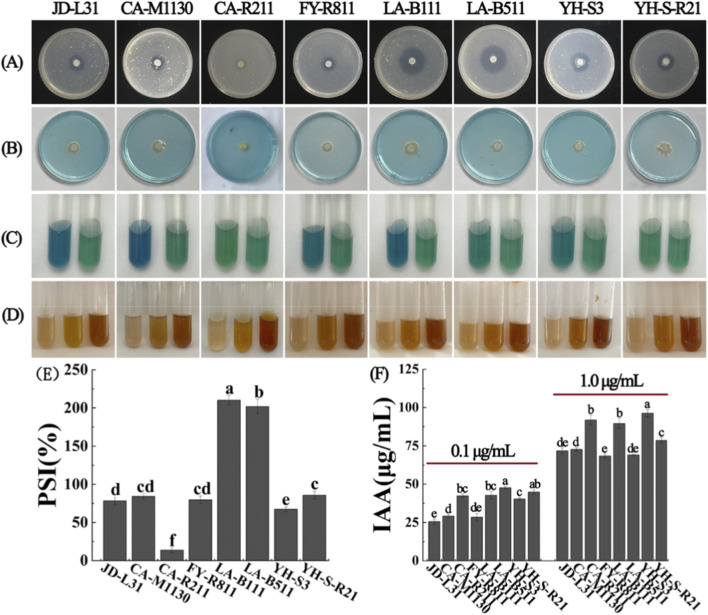
PGP trait characterization of eight bacterial strains. Transparent halos on National Botanical Research Institute’s phosphate (NBRIP) media after 3 days incubation at 28 °C, implying phosphorus solubilization ability **(A)**. Orange halos on CAS agar media after 3 days of incubation at 28 °C, indicating siderophore production capacity **(B)**. Color change in the test tubes containing N-free malate broth (Nfb) from a greenish shade to bluish shade after 2 days of incubation in a rotary shaker (180 rpm, at 28 °C), showing nitrogen fixation ability **(C)**. In all treatments, control is present on the right and strain is present on the left. Development of a pink-red color in the test tubes containing Luria-Bertani (LB) broth with 0.1% or 1.0% tryptophan after 2 days incubation in a rotary shaker (180 rpm, at 28 °C), indicating IAA production **(D)**. In each treatment, without tryptophan is on the left, with 0.1% tryptophan is in the middle, with 1.0% tryptophan is on the right. The phosphorus solubilization index (PSI) of eight strains of bacteria was assessed by inoculating them onto NBRIP medium and measuring the halo zone diameter and colony diameters after 3 days of incubation at 28 °C **(E)**. IAA production of eight bacterial strains in LB media supplemented with 0.1% (left) or 1.0% (right) tryptophan **(F)**. Bars followed by the different lowercase letters reveal the significance among different treatments (*p* < 0.05).

To assess potential PGP properties, all isolated bacterial strains were screened for various growth-promoting traits. Results showed that eight strains displayed phosphorus solubilization, siderophores production, nitrogen fixation and IAA synthesis abilities (Table 2; Figures 1A–D). The evaluation of the phosphorus solubilization activity of the tested strains was performed in NBRIP media after 3 days of incubation. Actually, all tested strains formed transparent solubilization halos, with strains LA-B111, LA-B511 and YH-S-R21 exhibiting the highest PSI values (210.19%, 201.91%, and 85.67%, respectively) (Figure 1E). Regarding the siderophore production test, all tested strains demonstrated siderophore secretion by exhibiting obvious orange halos around their colonies on CAS agar medium. Halo diameters varied significantly among strains (ranging from 8.28 to 10.57 mm; *p* < 0.05), with strains JD-L31, CA-M1130 and YH-S-R21 showing the strongest siderophore production capacity (10.57, 9.61, and 9.59 mm, respectively).

**TABLE 2:** Phosphorus solubilization, nitrogen fixation, siderophore production, and IAA synthesis by eight tested bacterial strains.

Treatment	Phosphorus solubilization (mm)	Siderophore production (mm)	IAA production (μg/mL)		Nitrogen fixation
			0.1% tryptophan	1.0% tryptophan	
JD-L31	13.53 ± 0.39 e	10.57 ± 0.91 a	25.61 ± 1.69 e	71.77 ± 1.96 de	*++*
CA-M1130	14.80 ± 0.31 d	9.61 ± 0.50 b	29.17 ± 1.10 d	72.72 ± 1.22 d	*++*
CA-R211	11.74 ± 0.36 g	9.00 ± 0.49 bc	42.33 ± 1.25 bc	91.84 ± 3.30 b	*-*
FY-R811	13.97 ± 0.39 e	8.36 ± 0.60 c	28.52 ± 2.41 de	68.39 ± 1.42 e	*++*
LA-B111	24.27 ± 0.51 b	8.28 ± 0.15 c	42.72 ± 2.57 bc	89.64 ± 3.33 b	*++*
LA-B511	24.82 ± 0.81 a	8.46 ± 0.40 c	47.67 ± 0.94 a	69.10 ± 0.56 de	*+*
YH-S3	12.93 ± 0.24 f	8.73 ± 0.34 c	40.39 ± 0.74 c	96.22 ± 2.49 a	*++*
YH-S-R21	16.39 ± 0.45 c	9.59 ± 1.09 b	44.79 ± 1.41 ab	78.56 ± 1.85 c	*-*

Distinct letters in each column indicate statistically significant differences among various treatments (*p* < 0.05). –, no nitrogen fixation activity; +, weak activity; ++, strong activity.

Meanwhile, all strains produced substantial amounts of IAA, with significant differences observed among strains in media supplemented with 0.1% or 1.0% tryptophan (*p* < 0.05; Figure 1F). IAA yields were generally higher in the 1.0% tryptophan treatment than in the 0.1% tryptophan treatment, consistent with the role of tryptophan as a precursor for IAA biosynthesis. In the presence of 0.1% tryptophan, the highest IAA production was observed in strain LA-B511 with 47.67 μg/mL, followed by strains YH-S-R21 (44.79 μg/mL) and LA-B111 (42.72 μg/mL). In contrast, at 1.0% tryptophan, the highest IAA production was observed in strain YH-S3 with 96.22 μg/mL, followed by strains CA-R211 (91.84 μg/mL) and LA-B111 (89.64 μg/mL). Furthermore, six bacterial strains (except strains CA-R211 and YH-S-R21) exhibited nitrogen-fixing activity, as indicated by a color change from a greenish shade to blueish shade after 2 days of incubation in Nfb medium, confirming their ability to convert atmospheric nitrogen into plant-available forms. Overall, these bacterial strains possessed stable and diverse PGP traits, showing strong potential for microbial fertilizer production in the future.

### Growth promotion on rice under controlled phytotron conditions

**FIGURE 2:**
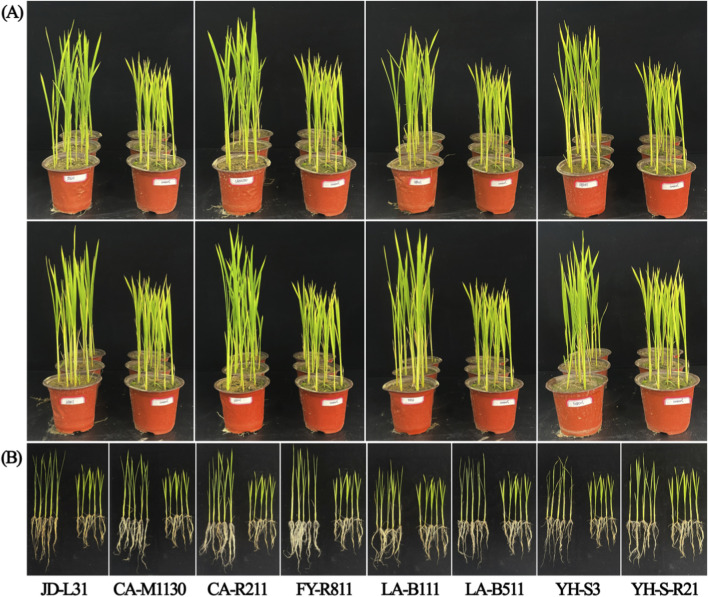
Impact of eight bacterial strains on rice growth in a phytotron at 35 days post-inoculation. All treatments included three replicates. Rice plants were inoculated with strains JD-L31, CA-M1130, CA-R211, FY-R811, LA-B111, LA-B511, YH-S3, and YH-S-R21, respectively **(A)**. Corresponding root samples are shown in **(B)**. For each treatment, the inoculated plants are on the left and the control plants are on the right.

To assess the influence of all eight bacterial strains on the growth of rice seedling in non-grain-cultivated farmlands, growth-promoting bioassays were carried out in a phytotron and plant growth was assessed following 35 days after inoculating tested bacteria (Table 3; Figure 2). Based on phenotypic observations, all eight bacterial strains displayed different degrees of promotional effects on rice growth. As well as, the measured data showed that all strains had significantly increased rice growth relative to control (*p* < 0.05). Among them, strains LA-B511, YH-S3, and LA-B111 were identified as the three most promising strains, showing the strongest growth-promoting effects on seedling height, root length, and dry biomass accumulation.

**TABLE 3:** Effect of eight bacterial strains on various rice growth parameters.

Treatments	SH (mm)	GPE%	RL (mm)	GPE%	SDW (mg)	GPE%	RDW (mg)	GPE%
JD-L31	185.60 ± 6.69	10.10 e	106.48 ± 4.26	−2.83 d	60.79 ± 3.92	1.37 d	31.14 ± 3.11	34.62 b
CA-M1130	207.39 ± 8.88	23.03 ab	119.17 ± 6.45	8.76 c	51.82 ± 5.36	−13.59 e	22.55 ± 3.17	−2.53 d
CA-R211	196.53 ± 4.49	16.58 cd	120.33 ± 7.55	9.82 c	67.72 ± 3.52	12.92 c	27.08 ± 2.59	17.10 c
FY-R811	203.00 ± 7.34	20.42 bc	121.57 ± 6.44	10.95 bc	71.10 ± 8.95	18.56 bc	28.40 ± 3.91	22.78 bc
**LA-B111**	**200.37 ± 3.53**	**18.86 c**	**133.63 ± 7.34**	**21.95 a**	**80.22 ± 5.10**	**33.78 a**	**34.89 ± 5.87**	**50.84 a**
**LA-B511**	**211.19 ± 6.74**	**25.28 a**	**128.00 ± 5.60**	**16.81 ab**	**72.45 ± 3.69**	**20.81 b**	**29.41 ± 2.47**	**27.17 bc**
**YH-S3**	**210.56 ± 8.88**	**24.90 a**	**124.72 ± 5.49**	**13.82 bc**	**83.08 ± 5.37**	**38.55 a**	**29.08 ± 5.11**	**25.74 bc**
YH-S-R21	191.59 ± 11.67	13.65 de	112.55 ± 12.10	2.71 d	62.70 ± 4.45	4.56 d	19.50 ± 2.64	−15.69 d
Control	168.57 ± 9.51	–	109.58 ± 8.45	–	59.97 ± 3.75	–	23.13 ± 3.58	**–**

SH, seedling height; RL, root length; SDW, seedling dry weight; RDW, root dry weight; GPE, growth promotion efficacy. Within each column, treatments denoted with distinct letters are significantly different (*p* < 0.05). The three treatments with the best growth-promoting properties are shown in bold.

Specifically, strain LA-B511 had shown substantial positive effects on rice growth, with a remarkable increase of 25.28% in seedling height, as well as substantial increases of 16.81%, 20.81% and 27.17% in root length, seedling and root dry weight, respectively, compared to the control (*p* < 0.05). Likewise, strain YH-S3 significantly promoted rice growth that resulted in 13.82% improvement of root length and 25.74% improvement of root dry weight, along with notable increases of 24.90% in seedling height and 38.55% in seedling dry weight compared to control (*p* < 0.05). In comparison with control, strain LA-B111 induced a notable positive impact on rice growth, with significant increases of 21.95%, 33.78% and 50.84% in root length, seedling and root dry weight, respectively, along with a substantial increase of 18.86% in seedling height, respectively (*p* < 0.05). Overall, current findings of the growth-promoting assays demonstrated the beneficial effects of the tested strains on growth of rice seedings in non-grain-cultivated farmlands. Notably, the three most promising strains (LA-B511, YH-S3, LA-B111) displayed superior and stable growth-promoting capabilities, further highlighting their potential as high-efficiency microbial fertilizers for sustainable rice production in non-grain-converted soil environments.

### Bacterial identification

To identify the eight strains, 16S *rRNA* sequences of each strain were blasted against GenBank database using BLASTn. Results showed that five strains (CA-M1130, FY-R811, LA-B111, LA-B511, and YH-S-R21) were intimately associated to species of *Enterobacter* having 99.93%, 99.71%, 100%, 100%, and 100% similarities with *Enterobacter asburiae* strain 10B, *Enterobacter cloacae* strain B, *Enterobacter hormaechei* strain Eh_11, *E. hormaechei* strain Eh_11, and *E. cloacae* strain PB-S1, respectively. Strains CA-R211, JD-L31, and YH-S3 were intimately associated to species of *Pantoea*, *Pseudonas*, and *Yokenella* having 100% similarities with *Pantoea ananatis* strain T38, *Pseudonas mosselii* strain CIFRI.S1, and *Yokenella regensburgei* strain CTRTIPSB22-4, respectively.

**FIGURE 3:**
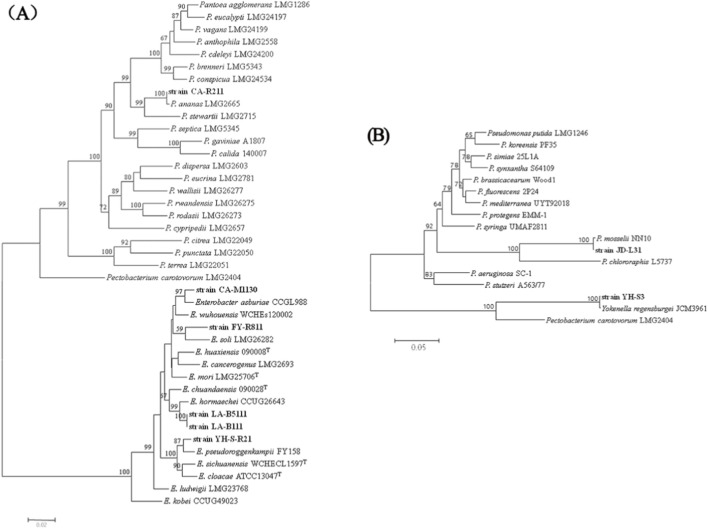
A maximum likelihood (ML) phylogenetic analysis was done based on the combined 16S *rRNA*, *gyr*B, *inf*B, *rpo*B, *atp*D **(A)** or 16S *rRNA*, *gyr*B, *rpo*B **(B)** genes sequences obtained from six independent sampling sites with diverse land-use conversion modes. Reference sequence of relevant taxa was retrieved from the GenBank and soil-derived strains obtained in this study are indicated in bold. Bootstrap values, calculated from 1,000 replicates, are shown as percentages.

To accurately identify the species of *Pantoea* and *Enterobacter*, a phylogenetic tree was made based on the sequences of the 16S *rRNA*, *gyr*B, *inf*B, *rpo*B and *atp*D genes. The phylogenetic tree showed that six strains (CA-R211, CA-M1130, FY-R811, LA-B111, LA-B511, and YH-S-R21) were separated into five different clades (Figure 3A). The strains CA-M1130 and FY-R811 clustered with *E. asburiae* strain CCGL988 and *Enterobacter soli* strain LMG26282 into a clade with a bootstrap of 97% and 59%, suggesting strains CA-M1130 and FY-R811 can be classified as *E. asburiae* and *E. soli*, respectively. Strains LA-B111 and LA-B511 clustered together with *E. hormaechei* strain CCUG26643 into a clade with 99% bootstrap support, indicating these two strains can be classified as *E. hormaechei*. Strain YH-S-R21 clustered with *Enterobacter pseudoroggenkampii* strain FY158, forming a clade supported by an 87% bootstrap value, indicating that YH-S-R21 should be inferred as *E. pseudoroggenkampii*. Due to the clustering of strain CA-R211 with *P. ananati* strain LMG2665 as a clade with a bootstrap of 100%, it was identified as *P. ananati*. Similarly, to further identify the species of *Pseudonas* and *Yokenella*, a phylogenetic tree based on 16S *rRNA*, *gyr*B, and *rpo*B was carried out. The phylogenetic analysis revealed that strains JD-L31 and YH-S3 were placed in two clearly distinct evolutionary lineages (Figure 3B). More specifically, strain JD-L31 grouped with *P. mosselii* strain NN10, forming a clade supported by a 100% bootstrap value, indicating that JD-L31 can be assigned to *P. mosselii*. In contrast, strain YH-S3 clustered with *Y. regensburgei* strain JCM3961 with 100% bootstrap support, supporting its identification as *Y. regensburgei*.

### Improvement of soil quality and microbial communities by PGPB in non-grain-converted land

The three most promising PGPB strains LA-B511 (*Enterobacter hormaechei*), YH-S3 (*Yokenella regensburgei*), and LA-B111 (*E. hormaechei*), which exhibited the strongest growth growth-promoting characteristics in previous bioassays, were further chosen to analyze their impacts on soil microbes and quality after 35 days of inoculation in non-grain-converted land. Analysis of soil physicochemical properties showed that inoculation with these three strains had no significant effects on soil pH, SOM, TP, or Fe relative to control treatment (*p* < 0.05). In contrast, AP content was markedly improved in all three inoculation treatments as compared to control treatment (*p* < 0.05) (Table 4), exhibiting increases of 27.00%, 25.99%, and 16.65% in Eh-LAB511 (inoculated with strain LA-B511), Yr-YHS3 (inoculated with strain Yr-YHS3), and Eh-LAB111 (inoculated with strain LA-B111) treatments, respectively. Collectively, these findings demonstrate that the three promising PGPB strains can effectively enhance soil phosphorus availability, a key limiting soil nutrient for rice growth in non-grain-converted land.

**TABLE 4:** Impact of three PGPB strains on soil characteristics.

Treatments	pH	SOM (g/kg)	TP (g/kg)	AP (mg/kg)	Fe (mg/kg)
Eh-LAB511	8.28 ± 0.06 a	7.10 ± 0.34 a	0.74 ± 0.05 ab	40.60 ± 1.09 a	25.98 ± 0.81 a
Yr-YHS3	8.25 ± 0.07 a	7.30 ± 0.75 a	0.67 ± 0.04 b	40.38 ± 1.08 a	25.43 ± 0.67 ab
Eh-LAB111	8.19 ± 0.08 a	7.22 ± 0.28 a	0.77 ± 0.04 a	37.29 ± 0.65 b	24.20 ± 0.41 b
**Control**	**8.15 ± 0.06 a**	**6.89 ± 0.49 a**	**0.70 ± 0.03 ab**	**31.97 ± 0.86 c**	**24.82 ± 0.74 ab**

^a,b,c^Within the same column, values marked with distinct letters differ significantly at *p* < 0.05.

SOM, denotes soil organic matter; TP, refers to total phosphorus; AP, indicates available phosphorus, and Fe represents total Fe; Eh-LAB511, treatment inoculated with strain LA-B511; Yr-YHS3, treatment inoculated with strain YH-S3; Eh-LAB111, treatment inoculated with strain LA-B111. The control is shown in bold.

**FIGURE 4:**
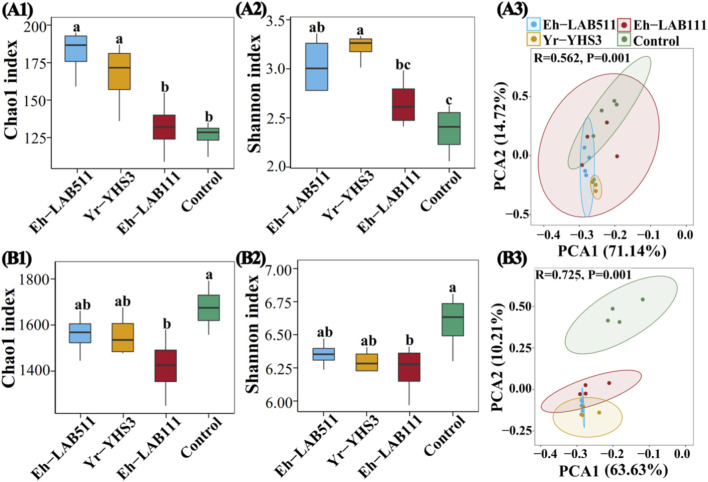
The impact of three distinct PGPB strains on the Chao1 and Shannon indices for fungi **(A1, A2)** and bacteria **(B1, B2)** is shown. Distinct letters indicate significant differences across various treatments (*p* < 0.05). Principal component analysis (PCA) results for the soil fungal **(A3)** and bacterial **(B3)** communities, with ellipses representing each treatment at a 0.95 confidence level**.**

To characterize the effects of the three promising PGPB strains on soil microbial communities, the fungal and bacterial community compositions and structures in soil samples were compared between treatments inoculated with strain LA-B511, YH-S3, or LA-B111 and the control, based on high-throughput amplicon sequencing. The average Chao1 index for fungi was 182 (159–195), 167 (136–187), 132 (109–155), and 126 (112–135), and the average Shannon index of fungi was 3.04 (2.78–3.36), 3.22 (3.01–3.33), 2.66 (2.42–2.99), and 2.38 (2.06–2.63) in Eh-LAB511, Yr-YHS3, Eh-LAB111, and control, respectively (Figures 4A1, A2). Generally, inoculation with the three PGPB strains increased fungal alpha diversity compared to control. Specifically, the Eh-LAB511 and Yr-YHS3 treatments led to significant increases in both Chao1 (44.26% and 32.14%, respectively) and Shannon indexes (27.83% and 35.49%, respectively) (*p* < 0.05), while the Eh-LAB111 treatment caused slight increases (4.71% in Chao1 and 11.97% in Shannon indices) without statistical significance.

In contrast, bacterial alpha diversity was slightly reduced by PGPB inoculation. The average Chao1 index for bacteria was 1,560 (1,445–1,661), 1,556 (1,478–1,676), 1,417 (1,246–1,577), and 1,675 (1,557–1,792), and the average Shannon index for bacteria was 6.35 (6.24–6.47), 6.30 (6.23–6.41), 6.24 (5.97–6.41), and 6.59 (6.30–6.81), respectively (Figures 4B1, B2). In other words, strains LA-B511, YH-S3, and LA-B111 inoculation significantly reduced the bacterial Chao1 index by 6.82%, 7.08%, and 15.39% (*p* < 0.05), and the bacterial Shannon index by 3.65%, 4.46%, and 5.43% (*p* < 0.05), respectively, compared to the control. Overall, the three PGPB strains differentially regulated soil microbial alpha diversity. Specifically, fungal richness and diversity were increased relative to the control, whereas bacterial richness and diversity were significantly reduced. Such distinct responses may reflect specific interactions between the inoculated PGPB strains and indigenous soil microbial communities in non-grain-converted land.

To assess differences and similarities in soil fungal and bacterial community structures across various treatments, PCA at OTU level was performed using the Bray-Curtis metric (Figures 4A3, B3). Particularly, OTU abundance in the 16 soil samples of Eh-LAB511, Yr-YHS3, Eh-LAB111 treatments and the control comprised four different groups. In detail, for fungal communities, there was noticeable overlap observed among Eh-LAB511, Eh-LAB111 treatments and the control, while Yr-YHS3 treatment was well separated from the control. The first principal component (PC1) explained 71.14% of the variance, while the second principal component (PC2) accounted for 14.72%, together representing approximately 86% of the total variance. In addition, all three inoculated treatments were all well separated from the control in the bacterial communities. PC1 and PC2 accounted for 63.63% and 10.21% of the total variation, respectively, meaning the two principal components together explained around 74% of the overall variance. A PERMANOVA analysis of the samples from all four treatments further indicated that the different strains explained 56.2% of the variance in fungal communities and 72.5% in bacterial communities (*p* = 0.01). In general, these results indicate that inoculation with strains LA-B511, YH-S3 and LA-B111 significantly reshaped bacterial community composition, whereas fungal community structure remained largely unaltered across most treatments, except following YH-S3 inoculation.

**FIGURE 5:**
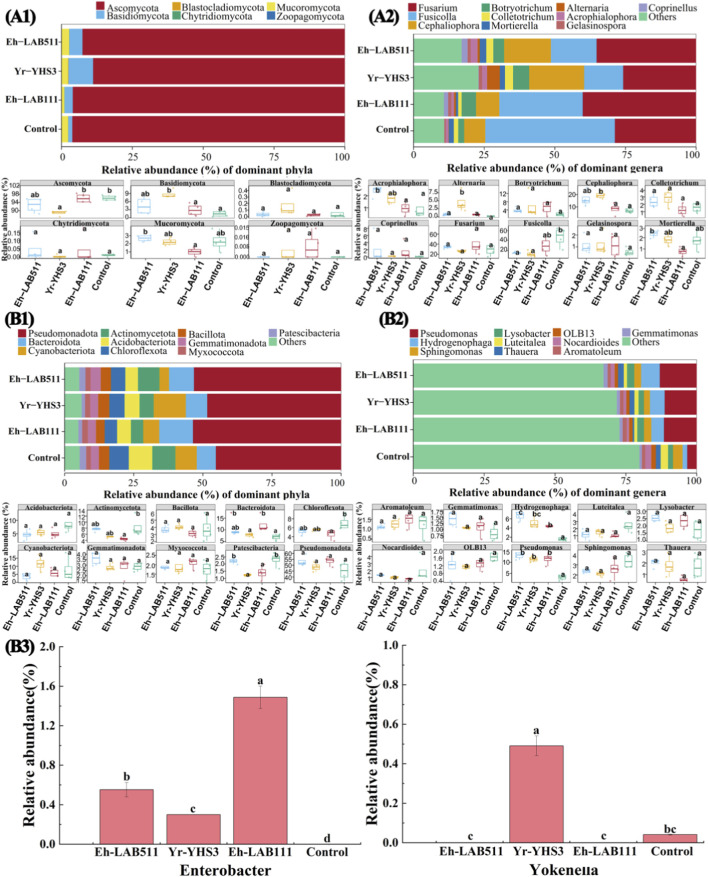
The relative abundance of fungi **(A)** and bacteria **(B)** at both phyla **(A1, B1)** and genera **(A2, B2)** levels, along with the abundance of bacterial biomarkers in genera across Eh-LAB511, YR-YHS3, Eh-LAB111 treatments and the control **(B3)**. Distinct letters indicate significant differences across various treatments (*p* < 0.05).

To better illustrate compositional differences in fungal and bacterial communities across the four treatments, histograms of RAs of top 10 phyla and genera were generated (Figure 5). Briefly, in fungal communities, Blastocladiomycota, Basidiomycota, and Ascomycota were the major phyla (Figure 5A1), and *Fusarium*, *Fusicolla*, *Cephaliophora*, *Botryotrichum*, and *Colletotrichum* dominated as the top genera (Figure 5A2). As compared to the control, the Eh-LAB511 treatment increased the RAs of *Fusarium* (22.35%), *Colletotrichum* (58.23%), *Botryotrichum* (83.49%), and *Cephaliophora* (120.23%), while significantly decreasing that of *Fusicolla* by 64.58% (*p* < 0.05). In the Yr-YHS3 treatment, the RAs of *Colletotrichum* (82.26%), *Cephaliophora* (159.27%, *p* < 0.05), and *Botryotrichum* (174.57%) were elevated, whereas those of *Fusarium* (10.21%) and *Fusicolla* (69.94%, *p* < 0.05) were reduced. Similarly, the Eh-LAB111 treatment increased the RAs of *Cephaliophora* (10.80%), *Fusarium* (39.37%), and *Botryotrichum* (142.58%), but decreased those of *Fusicolla* (35.54%) and *Colletotrichum* (22.23%). Meanwhile, based on RA and distribution of bacteria in four treatments, the predominant bacterial phyla were Pseudomonadota, Bacteroidota, Cyanobacteriota, Actinomycetota, Acidobacteriota, and Chloroflexota, with their RAs ranging from 36.39% to 59.67%, 5.26%–17.36%, 0.69%–18.40%, 3.77%–13.14%, 2.32%–12.92%, and 3.05%–9.35%, respectively (Figure 5B1). Furthermore, at the genus level, *Pseudomonas*, *Hydrogenophaga*, *Sphingomonas*, *Lysobacter*, *Luteitalea*, *Thauera*, *OLB13*, *Nocardioides*, *Aromatoleum*, and *Gemmatimonas* were dominated as top genera (Figure 5B2). As compared to control, the Eh-LAB511 treatment increased the RAs of *Thauera* (21.10%), *Lysobacter* (50.52%), *Gemmatimonas* (64.84%), *Pseudomonas* (287.44%, *p* < 0.05), and *Hydrogenophaga* (300.03%, *p* < 0.05), while decreasing those of *Aromatoleum* (9.48%), *OLB13* (27.10%), *Sphingomonas* (28.91%), *Nocardioides* (33.23%), and *Luteitalea* (55.38%), respectively. The Yr-YHS3 treatment enhanced the RAs of *Aromatoleum* (2.03%), *Thauera* (4.83%), *Lysobacter* (10.09%), *Gemmatimonas* (24.40%), *Hydrogenophaga* (211.39%), and *Pseudomonas* (237.78%, *p* < 0.05), but reduced those of *OLB13* (30.79%), *Sphingomonas* (37.21%), *Luteitalea* (43.60%), and *Nocardioides* (50.45%), respectively. The Eh-LAB111 treatment elevated the RAs of *Aromatoleum* (20.08%), *Gemmatimonas* (27.17%)*, Lysobacter* (36.92%), *Hydrogenophaga* (167.48%), and *Pseudomonas* (245.10%, *p* < 0.05), whereas lowered those of *OLB13* (22.73%), *Sphingomonas* (23.47%), *Luteitalea* (57.97%), *Nocardioides* (61.54%), and *Thauera* (64.73%), respectively. In particular, the RA of *Yokenella* in the Yr-YHS3 treatment were 11.93-fold of that in the control, while *Enterobacter* in the Eh-LAB511 and Eh-LAB111 treatments were 0.55- and 1.49-fold of that in the control, respectively (Figure 5B3). In conclusion, microbial community composition in the treatments inoculated with the three PGPB strains was significantly altered, with some microbes showing an increase in abundance while others exhibited a decrease as compared to control.

**FIGURE 6:**
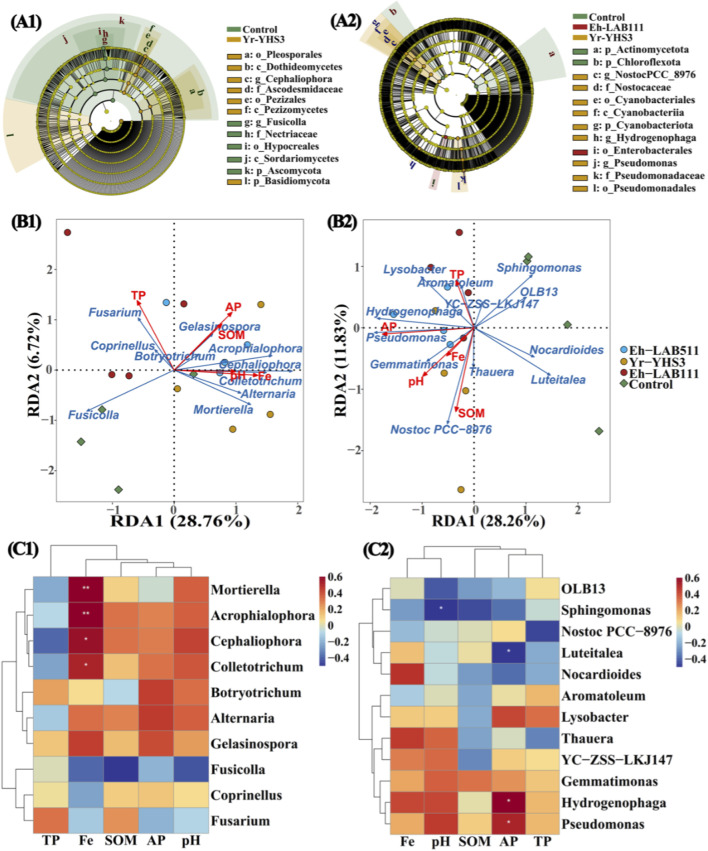
Linear discriminant analysis (LDA) effect size was used to identify fungal **(A1)** and bacterial **(A2)** taxa that differed significantly in abundance among the Eh-LAB511, YR-YHS3, Eh-LAB111 treatments and the control soils. Only taxa with an LDA score greater than 4.0 (*p* < 0.05) are presented. Redundancy analysis (RDA) was conducted to evaluate the relationships between soil physicochemical properties and fungal **(B1)** or bacterial **(B2)** communities at the genera level. In addition, clustering correlation heatmaps illustrating the associations between soil parameters and fungal **(C1)** or bacterial taxa **(C2)** across the four treatments are shown. SOM denotes soil organic matter, TP represents total phosphorus, AP indicates available phosphorus, and Fe refers to total iron.

Furthermore, LEfSe was performed for the identification of specific microbial biomarkers with remarkable differences distinguishing soil microbial communities among the Eh-LAB511, Yr-YHS3, Eh-LAB111 treatments and the control (Figure 6A). Results showed that 12 fungal biomarkers (LDA >4.0) and 12 bacterial biomarkers (LDA >4.0) were identified across all treatments. In brief, the communities in the Yr-YHS3 treatment were enhanced with Ascodesmidaceae, Basidiomycota, *Cephaliophora*, Dothideomycetes, Pezizales, Pezizomycetes, Pleosporales, as well as Cyanobacteriales, Cyanobacteriia, Cyanobacteriota, *Hydrogenophaga*, *NostocPCC_8976*, Nostocaceae, *Pseudomonas*, Pseudomonadaceae and Pseudomonadales. Communities of Eh-LAB111 treatment were enriched with Enterobacterales. The controls were enriched with Ascomycota, *Fusicolla*, Hypocreales, Nectriaceae, Sordariomycetes, Actinomycetota and Chloroflexota. Overall, these microbial taxa may have vital functions in maintaining the function and stability of soil microbial communities to promote plant growth.

Taking into account the impact of physicochemical properties of soil on microbial community composition, RDA was performed in this study (Figure 6B). Results of the current study represented that Fe, TP, AP, SOM and pH explained 41.25% (*p* = 0.028), 39.73% (*p* = 0.036), 39.36% (*p* = 0.043), 26.04% (*p* = 0.150) and 22.04% (*p* = 0.200) variation in the composition of the fungal community at the genus level, respectively, with axis 1 (28.76%) and axis 2 (6.72%) together explaining 35.48% of the total variation. Meanwhile, AP, SOM, pH, TP and Fe explained 82.97% (*p* = 0.001), 49.75% (*p* = 0017), 45.46% (*p* = 0.022), 15.07% (*p* = 0.336) and 13.87% (*p* = 0.367) variation in composition of bacterial communities at genera level, respectively, with axis 1 (28.26%) and axis 2 (11.83%) accounting for 40.09% of total variation. At the same time, the clustering correlation heatmap with signs clearly demonstrated that soil physicochemical characteristics have significant effect on microbes at the genera level (Figure 6C). These findings represented that *Acrophialophora*, *Cephaliophora*, *Colletotrichum* and *Mortierella* were remarkably (*p* < 0.05) positively associated with Fe, whereas *Sphingomonas* was negatively interrelated with pH. *Hydrogenophaga* and *Pseudomonas* were positively associated with AP, while *Luteitalea* was negatively associated with AP. Thus, it can be inferred that Fe, TP and AP were identified as the key factors driving fungal community variation, whereas AP, SOM and pH were the main determinants of bacterial communities, indicating that soil physicochemical properties may have shaped the distribution of soil microbes at the genera level.

## Discussion

Food security always is a crucial strategic issue related to the economic development, social stability, and national independence (Liu et al., 2021). With socio-economic development, major shifts in dietary patterns are occurring, with the consumption of basic grains to more diversified and healthy diets (Su et al., 2024; Dai et al., 2025). To meet diverse demands, lots of croplands have been widely applied to a range of non-grain crops, including vegetables, fruits, and bamboo (Chen et al., 2023). According to the China Statistical Yearbook, as non-grain crop production increasing from 19.7% to 30.3% from 1978 to 2020, the national grain acreage decreased by 3,819 thousand hectares. Unregulated expansion of non-grain crops has led to various negative consequences, including jeopardizing food security by insufficient food supply, degrading soil quality, and creating environmental issues by altering practices of land usage (Cheng et al., 2022; Jiang et al., 2024). As we know, chemical fertilizers play a pivotal role in modern agricultural systems by providing essential nutrients such as nitrogen, phosphorus, and potassium to crops (Kaur et al., 2025). However, over the last few decades, the overuse of chemical fertilizers has already occurred in China and caused negative environmental impacts on plants, soil, water, and air (de Souza et al., 2015). Especially, long-term heavy fertilization has caused soil acidification, which in turn significantly decreases crop yield and product quality, reduces soil available nutrients and disrupts soil structure (Zhang et al., 2024).

To reduce these negative impacts, achieve maximum benefits, and promote sustainable agriculture, PGPB-based microbial fertilizers have particularly attracted significant attention (Wang et al., 2023). Over the past few decades, numerous PGPB strains encompassing a wide range of species, including *Achromobacter*, *Acinetobacter*, *Agrobacterium*, *Alcaligenes*, *Aneurinibacillus*, *Aquaspirillum*, *Azorhizobium*, *Azotobacter*, *Bacillus*, *Brevundimonas*, *Burkholderia*, *Caldicellulosiruptor*, *Citrobacter*, *Corynebacterium*, *Cupriavidus*, *Curtobacterium*, *Delftia*, *Dyadobacter*, *Ensifer*, *Enterobacter*, *Erwinia*, *Gluconacetobacter*, *Herbaspirillum*, *Klebsiella*, *Kushneria*, *Listonella*, *Lysinibacillus*, *Methylobacterium*, *Micrococcus*, *Myroides*, *Novosphingobium*, *Ochrobactrum*, *Paenibacillus*, *Pantoea*, *Planococcus*, *Planomicrobium*, *Proteus*, *Providencia*, *Pseudomonas*, *Rahnella*, *Ralstonia*, *Raoultella*, *Rhizobium*, *Rhodococcus*, *Serratia*, *Sporosarcina*, *Sphingobacterium*, *Staphylococcus*, *Stenotrophomonas*, *Streptoccoccus*, *Tsukamurella*, *Vibrio*, *Xanthobacter*, *Xanthomonas* (Kaur et al., 2025). Applying PGPB in agricultural systems provides an economical and environmentally friendly solution for improving soil quality and stimulating plant growth (Ajijah et al., 2023).

### Isolation, identification and characterization of PGPB and their effect on rice growth

To enhance soil quality and promote plant growth in non-grain-converted land, a series of experiments were performed to screen PGPB based on their multifarious PGP properties such as phosphorus solubilization, siderophore production, IAA synthesis, and nitrogen fixation in the present study. As an important plant nutrient, phosphorus is critically involved in the regulation of biochemical and physiological processes and contributes significantly to enhanced tolerance to abiotic stresses (Khan et al., 2023). Nevertheless, the predominant fraction of soil phosphorus occurs as insoluble inorganic or organic compounds, rendering it inaccessible for effective plant utilization (Tang et al., 2023). Novel phosphorus-solubilizing bacteria have the ability to convert insoluble phosphorus into plant-available forms through mechanisms such as organic acid secretion, proton excretion, and enzyme production (Chen et al., 2019). Initial screening in this present study showed that eight bacterial strains had a marked phosphorus-solubilizing activity on NBRIP medium as visualized by a clear zone formed around the colony. Among them, three most promising strains (LA-B511, YH-S3, and LA-B111) directly corresponded to significant increases in soil AP and rice biomass accumulation in our controlled phytotron assay, establishing a direct causal link between *in vitro* phosphorus-solubilizing capacity and *in planta* growth promotion. Interestingly, Khan et al. (2023) reported that the improved phosphorus availability induced by PGPB inoculation can effectively promote chlorophyll synthesis and enhance photosynthetic efficiency, thereby accelerating biomass accumulation in rice seedlings and providing a clear physiological explanation for the observed growth-promoting effects. Consistent with the findings of this study, bacterial species of *Pantoea*, *Pseudomonas* and *Enterobacter* have shown the potential to increase plant growth by phosphorus solubilization (Shahid et al., 2012; Mahanta et al., 2014; Qingwei et al., 2023) and improving plant responses to abiotic stress (Rajini et al., 2020).

Additionally, siderophore-producing bacteria have crucial roles in growth of plant *via* enhancing plant tolerance to salt stress and Fe deficiency. These bacteria chelate available forms of iron (Fe^3+^) in the rhizosphere soil, restricting access to iron by pathogenic microbes, and limiting their proliferation (Zhang et al., 2019; Sultana et al., 2021). Similarly, our findings confirmed that all eight bacterial strains possessed the ability to produce siderophores as visualized by forming an orange halo around the strains on CAS plates. In addition to promoting crop growth through siderophore production, PGPB can enhance Fe bioavailability in food crops, thereby improving micronutrient deficiencies in human nutrition (Kaur et al., 2020; Rana et al., 2021). Moreover, PGPB can also directly enhance plant growth by producing phytohormones, particularly IAA, which is primarily involved in regulating root growth (a key phenotypic effect of IAA activity) (Taghavi et al., 2009). As an example, bacterial production of IAA promoted root development in tomato (Emami et al., 2019). In our study, the IAA concentrations produced by the screened growth-promoting bacterial strains ranged from 25.61 to 96.22 μg/mL. The superior IAA yields of strains LA-B511, YH-S3, and LA-B111 were closely associated with significant increases in rice root length and root dry weight in our phytotron assay, indicating that IAA-mediated root system enhancement is another core mechanism driving the growth-promoting effects of these strains. In addition, nitrogen fixation ability is critical for bacterial survival and grown, with enormous beneficial effects on plants (Sultana et al., 2024). Our findings indicate that six of the eight screened strains could fix atmospheric nitrogen as visualized by a change in color of Nfb medium from greenish to bluish. In previous investigations, we screened and characterized multiple soil bacterial isolates with nitrogen-fixation potential from farmland soils (Ali et al., 2021; Li et al., 2021). Overall, PGPB generally promote plant growth through direct (increasing nutrient availability to influence soil fertility through nitrogen fixation, mineral nutrients solubilization, organic compounds mineralization, and regulating various phytohormone production) and indirect mechanisms (suppressing a broad range of pathogens and protecting plants from adverse health effects) (Ajijah et al., 2023; Bashir et al., 2024). Interestingly, although all eight screened strains exhibited high degrees of growth-promoting characteristics, their performance in promoting rice growth varied in *in vivo* assays. For instance, strain YH-S3 greatly promoted rice seedling growth in bioassays, despite not demonstrating strong siderophore or IAA production in *in vitro* tests. The inconsistencies within *in vitro* and *in vivo* outcomes might be attributed to the differing biological properties of the bacterial metabolites, variations in the ability of colonization of different bacteria in soil, or various abilities to interact with plant. On the whole, the growth-promotion of rice seedlings was strain-dependent, and strains LA-B511, YH-S3, and LA-B111 exhibited the optimal growth-promoting effects under our controlled experimental conditions.

Depending upon the phylogenetic analysis of multiple bacterial gene sequences, all eight bacterial strains were classified into seven species: *P. ananas, E. asburiae, Enterobacter soli, Enterobacter hormaechei, Enterobacter pseudoroggenkampii, P. mosselii and Y. regensburgei*. Earlier research has demonstrated that some of these strains possess promising applications in agriculture. For example, Li Y. et al. (2024) found that under alkali stress, the lengths and dry weights of *Medicago sativa* after *E. asburiae* A103 inoculation improved by 21.9%–42.9% and 35.9%–37.1%, respectively. Tomato seeds treated with *E. hormaechei* exhibited increased biomass and shoot length compared to the control (Ranawat et al., 2021). Sokolova et al. (2025) reported that *E. soli* strain AF-22b-4245 had positive impact on wheat growth. Although effects of different *P. ananatis* strains ranged from growth promotion, to no effect, to pathogenic (Sheibani-Tezerji et al., 2015), bioassays revealed potent PGP properties when *P. ananatis* inoculated in cucumber, sorghum, and duckweed treatments (Meepagala et al., 2024). Egamberdieva (2012) showed that *Pseudomonas chlororaphis* strain TSAU13 could increase tomato shoot growth, dry matter, and fruit yield by 32%, 43%, and 16%, respectively, compared to the control. *P. mosselii* strain PR5 upregulated the growth and biomass of rice, with maximum shoot and root fresh weight increased by 49.3% and 33.5%, respectively, when compared with control (Sultana et al., 2021). Consistent with previous reports, the eight strains in this study significantly increased rice seedling height by 10.10%–25.28%, root length by 2.71%–21.95% (except a 2.83% decrease by JD-L31), shoot dry weight by 1.37%–38.55% (except a 13.59% decrease by CA-M1130), and root dry weight by 17.10%–50.84% (except a 2.53% and 15.69% decrease by CA-M1130 and YH-S-R21, respectively) under controlled conditions. Notably, the application of *Y. regensburgei* as PGPB in agriculture is still limited.

### Change of soil properties and microbial communities by PGPB contributes to rice growth

Across eight PGPB strains, strains LA-B511 (identified as *E. hormaechei*), YH-S3 (identified as *Y. regensburgei*) and LA-B111 (identified as *E. hormaechei*) exhibiting the strongest growth-promoting traits were short-listed for detailed investigation. These findings described that inoculating plants with strains LA-B511, YH-S3 and LA-B111 has led to varying degrees of improvement on soil nutrients, in particular, the AP (37.29–40.60 mg/kg) was significantly increased by all three strains compared to the control (31.97 mg/kg). This result directly validates our *in vitro* phosphorus-solubilization assays, confirming that these strains retain their phosphorus-mobilizing capacity in non-grain-converted soil, with direct benefits for soil phosphorus availability and rice nutrient uptake. Consistent with current findings, phosphorus is a critical macronutrient involved in numerous physiological processes in plants, whereas PGPB inoculation could elevate soil AP in agroecosystems. For instance, Dobrzyński et al. (2025) reported that inoculation of a non-native PGPB consortium comprising *Pseudomonas* sp. G31 and *Azotobacter* sp. PBC2 significantly enhanced AP compared with the control treatment (48.03 mg/kg vs*.* 40.23 mg/kg). Esitken et al. (2010) found that AP contents were increased from 0.35 kg P_2_O_5_/da to 1.82–2.00 kg P_2_O_5_/da by *Pseudomonas* BA-8, *Bacillus* OSU-142 and *Bacillus* M-3 applications in strawberry soil. Although agricultural soils generally contain substantial amounts of phosphorus, only a limited fraction of inorganic phosphorus is readily available for plant uptake, as most exists in immobile and inaccessible forms. This limited bioavailability severely constrains crop productivity and agricultural development in many regions worldwide. Therefore, the mobilization and mineralization of unavailable phosphorus into plant-available forms represent a fundamental strategy for achieving agricultural sustainability (Zhang et al., 2021; Wang et al., 2024). Accordingly, the three strains (strains LA-B511, YH-S3 and LA-B111) are likely to play a significant role in improving soil quality (particularly soil phosphorus availability) in non-grain farmland converted from vegetable cultivation.

According to previous research, the importance of applying PGPB in agricultural fields depends mostly on their ability of colonizing, survival and effective adaptation in environment (Ajijah et al., 2023). Once an association established, PGPB can significantly reshape soil microbial community structure, thereby improving soil fertility, ecological functions, and crop productivity (Cheng and Ma, 2025). In fact, after 35 days of inoculation under controlled phytotron conditions, strains LA-B111, LA-B511 (*p* < 0.05) and YH-S3 (*p* < 0.05) significantly increased the fungal alpha diversity. Conversely, the richness of bacterial communities was noticeably reduced by strains LA-B111 (*p* < 0.05), LA-B511 and YH-S3. In all treatments, Ascomycota, Basidiomycota, Blastocladiomycota, *Fusarium*, *Fusicolla*, *Cephaliophora*, *Botryotrichum* and *Colletotrichum* were the main fungi, while Pseudomonadota, Bacteroidota, Cyanobacteriota, Actinomycetota, Acidobacteriota, Chloroflexota, *Pseudomonas*, *Hydrogenophaga*, *Sphingomonas*, *Lysobacter*, *Luteitalea*, *Thauera*, *OLB13*, *Nocardioides*, *Aromatoleum* and *Gemmatimonas* were the main bacteria. Furthermore, LEfSe also obtained 12 fungal biomarkers and 12 bacterial biomarkers in all treatments. In this study, inoculation with PGPB led to the enrichment of several fungal taxa, such as Basidiomycota (increase by 102.88%–507.00%), *Acrophialophora* (4.20%–158.00%), *Botryotrichum* (83.49%–174.57%), *Cephaliophora* (10.80%–159.27%), *Gelasinospora* (91.91%–106.43%), and some bacteria including Bacteroidota (12.96%–77.38%), Pseudomonadota (6.85%–1825%), *Gemmatimonas* (24.40%–64.84%), *Hydrogenophage* (167.48%–300.03%), *Lysobacter* (10.09%–50.52%), *Pseudomonas* (237.78%–287.44%). The enrichment of these nutrient-cycling and growth-promoting taxa was closely correlated with improved soil AP and enhanced rice growth, demonstrating that rhizosphere microbial community reshaping serves as another key mechanism underlying the PGP effects of the inoculated strains.

In line with current findings, Basidiomycota are known for their ability of degrading crop residues, owing to their complementary enzyme pools that enable the efficient decomposition of complex organic matter (Manici et al., 2024). *Acrophialophora jodhpurensis* isolate Msh5 can promote tomato plant growth and control the causal agent of tomato early blight, highlighting its dual potential in plant growth promotion and disease control (Daroodi et al., 2022). *Botryotrichum* produce many secondary metabolites involved in multiple agricultural applications, including plant growth regulation and pathogen inhibition (Elkhateeb and Daba, 2022). *Cephaliophora* can cause leaf spot on tomato, indicating the need to monitor their abundance in agricultural systems (Zhao et al., 2023). Several species of *Gelasinospora* have antagonistic effects against plant pathogenic fungi *in vitro*, suggesting their potential as biocontrol agents (Piasai and Sudsanguan, 2018). Bacteroidota are ecologically important carbohydrate degraders in soil and some species classified as PGPB in this phylum can antagonize various plant pathogens in different crops (Kruczyńska et al., 2023). Pseudomonadota play a widespread role in global ecosystems, particularly in nutrient cycling, including carbon, nitrogen, and phosphorus turnover (Ge et al., 2025). *Gemmatimonas* can involve N_2_O reduction in agricultural soils, which is vital for mitigating greenhouse gas emissions and maintaining soil ecological stability (Oshiki et al., 2023). *Hydrogenophage* can produce biosurfactants and degrade high molecular weight-polycyclic aromatic hydrocarbons, underscoring their potential in soil remediation (Yan et al., 2017). *Lysobacter* can produce a range of extracellular enzymes and other metabolites with activity against fungi, bacteria, and nematodes (Expósito et al., 2015). *Pseudomonas* can not only improve plant growth by synthesizing growth-stimulating plant hormones and suppressing pathogenic microbes, but also lessen the global dependence on harmful agricultural chemicals, contributing to sustainable agriculture (Bhardwaj et al., 2024). Obviously, compared to the control, all these microbes enriched in the Eh-LAB111, Eh-LB511 and Yr-YHS3 treatments may possess strong potential for successful colonization and persistence in rice fields converted from vegetable fields. Their enrichment is expected to improve soil physicochemical conditions, enhance nutrient bioavailability (particularly phosphorus), and reduce rice disease incidence, the key factors supporting soil health and rice growth in non-grain converted soils under the tested controlled conditions.

In addition, it was important to mention that RAs of *Yokenella* in the Yr-YHS3 treatment were 11.93-fold of that in the control, while *Enterobacter* in the Eh-LAB111 and Eh-LAB511 treatments were 1.49- and 0.55-fold of that in the control. According to previous studies, numerous *Enterobacter* strains can express a wide range of PGP activities to promote plant growth, suppress soil borne plant pathogens, enhance soil nitrogen content, and boost soil microbial functional-diversity (Jha et al., 2011; Chakraborty et al., 2019). *Yokenella* present a novel and promising avenue for addressing plastic pollution by degrading polystyrene (Toc et al., 2024), but their application in agricultural system is still limited. Furthermore, RDA and correlation-based clustering heatmaps revealed strong associations between soil properties and specific microbial taxa. Fe showed significant positive relationships with *Acrophialophora*, *Cephaliophora*, *Colletotrichum* and *Mortierella*, whereas soil pH exhibited a significant negative association with *Sphingomonas*. In addition, AP was positively associated with *Hydrogenophaga* and *Pseudomonas*, but displayed a significant negative correlation with *Luteitalea*. Consistent with our findings, Ragot et al. (2016) suggested that pH and associated soil factors were significant dominants of microbial and *phoD*-harboring community structures. The structure of rhizosphere microbial communities responds strongly to optimized phosphorus management under farming conditions (Deng et al., 2021). Sun et al. (2024) demonstrated that soil microbial community structure was strongly influenced by pH, total nitrogen, ammonium nitrogen, available potassium, available phosphorus, soil salinity, and organic carbon. Moreover, bacterial communities exhibited greater sensitivity to environmental variables than fungal communities during mangrove forest restoration. Overall, the application of the three PGPB strains substantially reshaped soil microbial communities by altering the abundance of specific microbial taxa. Changes in soil physicochemical properties likely played a critical role in driving these community shifts, thereby regulating rice growth.

Taken together, our findings carry substantial practical implications for the sustainable management of non-grain-converted lands and the development of eco-friendly agricultural systems across China. The three elite PGPB strains (LA-B511, YH-S3, and LA-B111) characterized in this study possess multiple complementary and functionally validated PGP traits, rendering them highly promising candidates for developing both single-strain inoculants and synergistic composite microbial fertilizers. Critically, these strains are specifically adapted to the degraded non-grain-converted lands characterized by widespread nutrient imbalance, soil acidification, and impaired indigenous microbial communities, thus filling a critical gap in targeted microbial amendments for these degraded agricultural soils. From an industrial and policy standpoint, the ability of these strains to significantly elevate soil AP and boost rice growth even under reduced chemical fertilizer input offers a practical, cost-effective strategy to alleviate agricultural over-reliance on synthetic fertilizers. This strategy directly aligns with China’s national strategic priorities of safeguarding food security, regulating non-grain conversion of land, and advancing green sustainable agriculture. These findings highlight strong application potential of these strains under controlled phytotron conditions. However, it would have been valuable to assess rice growth performance and rhizosphere microbial dynamics under natural field conditions using the selected PGPB strains owing to that microbial interactions and plant-soil feedbacks are often constrained under controlled phytotron conditions. Furthermore, future studies should be also carried out to systematically measure photosynthetic characteristics and nutrient uptake dynamics, which will help us better clarify the physiological mechanisms underlying PGPB-mediated rice growth promotion.

## Conclusion

In this study, eight PGPB strains isolated from soils representing six distinct non-grain cultivation systems demonstrated strong functional capacities, including phosphorus solubilization, siderophore production, IAA synthesis, and nitrogen fixation, although the strength of these activities differed among individual strains. Based on morphological characterization and phylogenetic analysis, all eight bacterial strains were classified into seven species, including *P. ananas*, *E. asburiae*, *E. soli*, *E. hormaechei*, *E. pseudoroggenkampii*, *P. mosselii*, and *Y. regensburgei*. Notably, inoculation with *E. hormaechei* and *Y. regensburgei* significantly promoted rice growth in non-grain-converted soil under controlled phytotron conditions. After 35 days of inoculation, soil physicochemical and microbial community analyses confirmed the potential of these strains as PGPB-based bio-organic fertilizers and bio-amendments for restoring soil quality and enhancing plant growth in non-grain-converted lands. As all experiments were conducted under controlled phytotron conditions to minimize environmental interference, the performance of these strains on rice growth and grain yield as well as their growth-promoting mechanisms should be further explored under natural field conditions. However, these results of this study clearly indicated that the screened PGPB strains have great potential in enhancing rice growth and soil health in the specific marginal non-grain-converted cultivated lands.
